# Characterization of the complete chloroplast genome of *Leucomeris decora* Kurz (Asteraceae)

**DOI:** 10.1080/23802359.2021.2008826

**Published:** 2021-12-19

**Authors:** Qinghua Li, Man Yang, Yue Lv, Yaqi Lv, Tian Li, Shilong Wang, Yonggui Ma, Yuhua Shi

**Affiliations:** a School of Life Sciences, Zhengzhou University, Henan, Zhengzhou, P. R. China; b Lab of Environment and Resource in Qinghai-Tibet Plateau, Ministry of Education, Qinghai Normal University, Qinghai, Xining, P. R. China

**Keywords:** *Leucomeris decora*, whole chloroplast genome, phylogenetic studies

## Abstract

*Leucomeris decora* is a traditional medicinal plant that is listed as an endangered species in China. Recently, *L. decora* has become locally rare. Here the complete chloroplast genome of *L. decora* was assembled and reported for the first time. Its plastome was 151,491 bp in length, including a large single-copy region (LSC; 83,155 bp), a small single-copy region (SSC; 18,216 bp), and a pair of inverted repeated regions (IRa and IRb; 25,060 bp). The overall GC content was 37.8%, and the genome contains 134 genes, including 92 protein-coding genes, 8 rRNA genes, and 34 tRNA genes. Phylogenetic analysis of thirteen representative species from the family of Asteraceae showed that *L. decora* is clustered into one clade with *Gerbera jamesonii* with high bootstrap values, indicating a close relationship between these two species.

*Leucomeris decora* Kurz belongs to the family Asteraceae, which is generally distributed in Southwest China (Funk et al. 2009; Zhao and Gong 2012). The plant’s leaves and stems have been used as a traditional anti-inflammatory medicine (Liu et al. 2018). Due to rapid habitat fragmentation and unrestricted utilization, *L. decora* has become endangered with a drastically reduced population size, and it is now listed in the IUCN Red List of Threatened Species and the Red Book of Chinese Plants (Fu and Jin 1992).

There is no clear morphological divergence between *L. decora* and other woody species of Asteraceae, such as *Nouelia insignis* Franch. The bisexual corolla of *Leucomeris* is radially symmetrical, and the bisexual corolla of *Nouelia* is symmetrical on both sides (Peng et al. 2002). Both of these genera possess the same number of chromosomes with 2n = 54, which differs from that in other genera of Asteraceae (Panero and Funk 2008; Zhao and Gong 2015). Despite differences in their floral traits, the genera of *Leucomeris* and *Nouelia* are clustered into a single group based on morphological and molecular evidence (chloroplast DNA and nuclear ribosomal DNA) (Zhao and Gong 2015). Although the taxonomic status of the genus *Leucomeris* has been elucidated to some extent, few studies have focused on the phylogenetic and adaptive evolution of this genus at the plastomic level.

Fresh leaves of *L. decora* were collected from Yunnan, China (N 24.943°, E 98.868°), and a specimen of *L. decora* (specimen No. 622) was deposited at the Herbarium, Kunming Institute of Botany, Chinese Academy of Sciences (under the voucher No. 622, Dr. Zhao, zhaoyujuan@mail.kib.ac.cn). Total genomic DNA was extracted from leaves using the modified CTAB method (Suzana et al. 2015) and NanoDrop 2000 spectrophotometer was used for determined the concentration of DNA. The sequenced library was constructed and generated with a paired-end library of 2 × 250 bp using Illumina HiSeq X platform (Illumina, Inc., America) with the modified manuscript. The genomic DNA were sequenced more than 6 GB raw data through the Illumina platform at Novogene (Beijing, China). All raw reads were trimmed and assembled with the GetOrganelle software (Jin et al. 2020). The chloroplast genome was annotated with both PGA and GB2sequin (https://chlorobox.mpimp-golm.mpg.de/geseq.html) using the chloroplast genome of *Gerbera jamesonii* Adlam (Genbank accession No. MN087227) as the reference. The tRNAscan-SE program was employed to identify tRNA genes with default parameters. A gene map of the annotated *L. decora* genome was drawn using the online tool OGdraw (https://chlorobox.mpimp-golm.mpg.de/geseq.html). Finally, the whole chloroplast genome of *L. decora* was submitted to GenBank under the accession No. MT386593.

The chloroplast genome of *L. decora* is 151,491 bp in length and exhibits a typical quadripartite structure: the two inverted repeat (IR) regions of 25,060 bp were separated by a large single-copy region (LSC; 83,155 bp) and a small single-copy (SSC; 18,216 bp) region. The GC content of the chloroplast genome was 37.8%, and the GC content of the LSC, SSC, and IR regions were 35.4, 31.5, and 43.2%, respectively. Ninety-two protein-coding genes, 34 tRNAs, and 8 rRNAs were detected in the whole chloroplast genome sequence. Out of these 134 genes, there were 20 duplicate genes (9 protein-coding genes, 7 tRNAs, and 4 rRNAs) in the IR regions and 20 genes which contained introns.

The whole chloroplast genome sequences of 13 species were downloaded to analyze the phylogenetic relationship among *L. decora* and other species of Asteraceae. *Campanula takesimana* Nakai (Genbank accession No. MW013763) in the family *Campanulaceae* was used as the outgroup. Alignment was conducted using MAFFT (Katoh and Standley 2013). Phylogenetic analysis was carried out based on maximum likelihood analysis by IQ-TREE 1.5.5 software (Nguyen et al. 2015), with TVM + F+R2 as the best nucleotide substitution model and 10,000 bootstrap replicates. The results of phylogenetic analysis showed that *L. decora* was close to *Gerbera jamesonii* Bolus, as reported by Fu et al. (2016), which supports that the chloroplast genome of *L. decora* provides useful genetic information for further studies on the genetic diversity and conservation of Asteraceae species (Figure 1).

**Figure 1.**
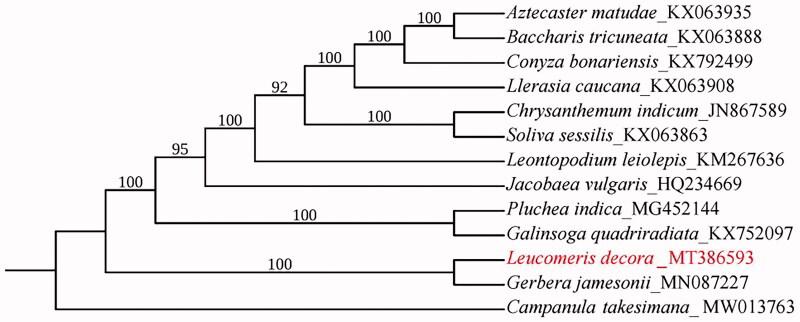
Maximum-likelihood phylogenetic tree for *L. decora* based on 13 complete chloroplast genomes. The number on each node indicates the bootstrap support value.

## Data Availability

The genome sequence data that support the findings of this study are openly available in GenBank of NCBI at [https://www.ncbi.nlm.nih.gov] under the accession no. MT386593. The associated Bioproject, SRA, and Bio-Sample numbers are PRJNA729624, SRR14520563, and SAMN19136515, respectively.
